# Visual perspective-taking ability in 7- and 12-month-old infants

**DOI:** 10.1371/journal.pone.0263653

**Published:** 2022-02-16

**Authors:** Ayaka Ikeda, Yasuhiro Kanakogi, Masahiro Hirai

**Affiliations:** 1 Center for Development of Advanced Medical Technology, Jichi Medical University, Tochigi, Japan; 2 Department of Psychology, Senshu University, Kanagawa, Japan; 3 Department of Comparative and Developmental Psychology, Graduate School of Human Sciences, Osaka University, Osaka, Japan; 4 Department of Cognitive and Psychological Sciences, Graduate School of Informatics, Nagoya University, Nagoya, Japan; 5 Department of Pediatrics, Jichi Medical University, Tochigi, Japan; University of Queensland, AUSTRALIA

## Abstract

Understanding how we see the world is different from how other people see it is a crucial part of social cognition and is known as visual perspective-taking. Although previous studies have demonstrated that 14-month-old infants have the capacity to compute the visual perspectives of others, it remains unknown whether infants under 12 months also have this ability. In this study, we modified a conventional gaze-following experimental setting in which one toy was placed in front of a model and was hidden by a barrier (Blocked Line of Sight Condition), and another toy was placed without a barrier (Clear Line of Sight Condition). We examined the visual perspective-taking abilities of 48 infants aged 7 and 12 months by measuring the infants’ gaze shift towards the gaze-cued toy object with and without a barrier. The results demonstrated that 12-month-old infants could correctly follow a model’s gaze if the model’s line of sight was clear. In contrast, 7-month-old infants showed no evidence of such capacity. Our findings indicate that 12-month-old infants seem to have the capacity to compute the visual perspective of others.

## Introduction

Computing what others see and think is a crucial ability to navigate the social world [1]. The ability to understand that other people have a different line of sight than we do is known as “visual perspective-taking” [2]. The cognitive and neural mechanisms underlying visual perspective-taking have already been revealed [3, 4] and theorized [5, 6] in previous studies. Theoretically, there are at least two levels of visual perspective-taking: Visual Perspective-Taking Level 1 (VPT1) and Level 2 (VPT2) [7]. VPT1 refers to the ability to infer whether another person can see an object or scene. VPT2 refers to the ability to infer whether other people see an object or scene differently from how we see it.

Developmental studies have demonstrated that these two levels of visual perspective-taking are not acquired simultaneously. A study conducted on children aged four to eight years showed that the VPT2 ability generally emerged between the ages of four and five [6, 8, 9] and improved considerably between the ages of six and eight [10–12] or later [13]. Moreover, a recent study showed that some three-year-old children were able to complete a VPT2 task successfully through social interaction with an experimenter [14].

In contrast, VPT1 development begins much earlier than VPT2. Research has shown that infants begin to employ VPT1 at approximately 24 months [15] or earlier [16–18]. The premise for computing others’ perspectives is to understand the function of the eyes for “seeing.” In a study based on the gaze-following paradigm, Brooks and Meltzoff directly manipulated the adults’ eye region with a blindfold to determine whether infants can understand how adults’ eyes can influence the function of sight [19]. When the experimenter’s eyes were open, 12-, 14- and 18-month-old infants followed their gaze more than when the model’s eyes were closed. Moreover, 14- and 18-month-old infants followed their gaze more in the headband condition as compared to the blindfold condition. However, 12-month-old infants did not show any conditional modulation for either condition. This suggests that 14- and 18-month-old infants did not simply respond to adults who turned their heads but seemed to interpret the role of eyes in seeing. Around the same time as the infants begin to understand “seeing,” they may also begin to understand others’ seeing behavior, such as VPT1. Early studies manipulated an adult’s view by placing a barrier in front of them. In this manipulation, infants could see both toy objects, but the adult could see only one toy object that was not occluded by the barrier. These studies demonstrated that 14-month-old infants seemed to understand that the experimenter could not see the toy object when the barrier was placed in the adult’s line of sight [20, 21]. Based on the looking-time paradigm by using a goal-directed action paradigm [22], Sodian, Thoermer, and Metz [16] demonstrated that 14-month-old infants looked longer when a person reached for a new toy object when the previously reached toy object was visible to the person, than when it was not visible to the person. In contrast, this modulation of looking time was not observed in 12-month-old infants. This suggests that 14-month-old infants could rationalize that another person’s reach for a new object depends on that person’s line of sight [16].

Although these studies imply that behavioral performance related to VPT1 seems to emerge around the age of 14 months depending on experimental settings and procedures, several other recent studies have implied that younger infants have the capacity to process another person’s seeing behaviors. Using gamma-band oscillatory responses as electrophysiological markers to represent object processing, the oscillatory activity was modulated for the condition in which the object was occluded from the infant’s perspective and when it was occluded from the adult’s perspective [23]. This finding indicates that 8-month-old infants could process the object correctly from their own viewpoint and from the adult’s viewpoint. Additionally, Kovacs, Teglas, and Endress [24] found that 7-month-old infants’ looking behaviors were modulated by the mere presence of a social agent, indicating that the presence of an agent can trigger belief computations of others. Moreover, 8-month-old infants had the capacity to understand referential expectations and expected that the target was located at the end of the adult’s line of sight [25]. These findings raise the possibility that even infants younger than 14 months have the capacity to process another person’s visual experience.

The current study aimed to test whether 7- and 12-month-old infants could reliably perform VPT1. To this end, we slightly modified the previous gaze-following paradigms [26, 27] to manipulate an adult’s line of sight by introducing a barrier. Gaze-following is a phenomenon in which infants change their attentional focus in response to another’s head and eye movements toward an object. Gaze-following emerges at around 4 months in infants [28, 29] and is robustly found at around 6.5 months when ostensive signals, such as eye contact and infant-directed speech, accompany the model’s movements [26]. Furthermore, 11- to 12-month-olds could perfectly follow the model’s gaze [30–32]. In addition to gaze-following, the “joint attention” process [33] should be involved. “Joint attention” is a goal-oriented behavior that shares experiences with other people [34, 35]. This involves information processing from another person’s as well as one’s own perspective [30, 36, 37]. Past studies on joint attention have shown that it is particularly well developed by the age of 12 months [38–40].

Since (1) infants who are at least 7-month-old seem to process others’ visual experiences, referential expectations, and beliefs [23–25], and (2) 12-month-old infants can understand the referential nature of looking [27, 41], we focused on both 7-month-old and 12-month-old infants. To the best of our knowledge, no study has directly tested whether 7- and 12-month-old infants can reliably reflect VPT1 computation on online behavior such as gaze-following.

In this experiment, we measured infants’ eye movements to determine their ability to compute an adult’s visual experience [26, 32, 42]. We predicted that if the infant understands the other person’s visual experience, (1) they can follow an adult’s gaze, when the adult sees a target, from the adult’s perspective, and (2) infants would not follow an adult’s gaze, when the adult cannot see a target, from the adult’s perspective. In addition to the gaze-following measure, we performed an exploratory analysis of the first looking time [19] and the total looking time [27] to examine whether infants had a longer looking time for an unexpected event (i.e., the model looking at the toy object occluded by a barrier) compared to an expected event (i.e., the model looking at the toy object not occluded by a barrier) [43].

## Materials and methods

### Participants

The participants in this study were 24 healthy, born at full term, typically developing, 7-month-old infants (mean age = 201.7 days; range = 186–230 days; 11 girls and 13 boys) and twenty-four 12-month-old infants (mean age = 379.5 days; range = 361–408 days; 17 girls and 7 boys). Although 34 other infants participated (7-month-olds: *n* = 24, 12-month-olds: *n* = 10), their data were excluded from the final analysis for one of the following three reasons: (1) fussiness (7-month-olds: *n* = 2, 12-month-olds: *n* = 0); (2) they did not meet the inclusion criterion for eye movement, which is described in the “Test Phase” section (7-month-olds: *n* = 22, 12-month-olds: *n* = 9); or (3) they were not born at full term (7-month-olds: *n* = 0, 12-month-olds: *n* = 1). We determined a set sample size for the experiment (*N* = 24 per group) by performing a power analysis based on effect size (*d*) of 0.93 from a previous study that used similar procedures, materials, dependent variables [27], and a similar experimental paradigm [26, 44]. All the infants’ parents provided written informed consent for their children to participate in the study. The study protocol was approved by the ethics committee of Jichi Medical University (A17-118) and was conducted in accordance with the Declaration of Helsinki.

### Apparatus

A Tobii TX-300 eye tracker (Tobii Technology Inc., Stockholm, Sweden) with a 23-inch TFT monitor was used to record the infants’ eye movements. The sampling frequency was 120 Hz. Infants were seated on their parents’ laps, with their eyes approximately 60 cm from the monitor. A five-point calibration was performed prior to the recording. Stimulus presentation and analysis were conducted using the Tobii Studio (version 3.4.8.1348).

### Stimuli and procedure

We recorded the actions of a female model with a video camera (GZ-E107-B, Victor Company of Japan, Ltd.) placed in front of the model. The experiment was composed of three phases (Introduction Phase, Familiarization Phase, and Test Phase).

#### Introduction Phase

**Fig 1:**
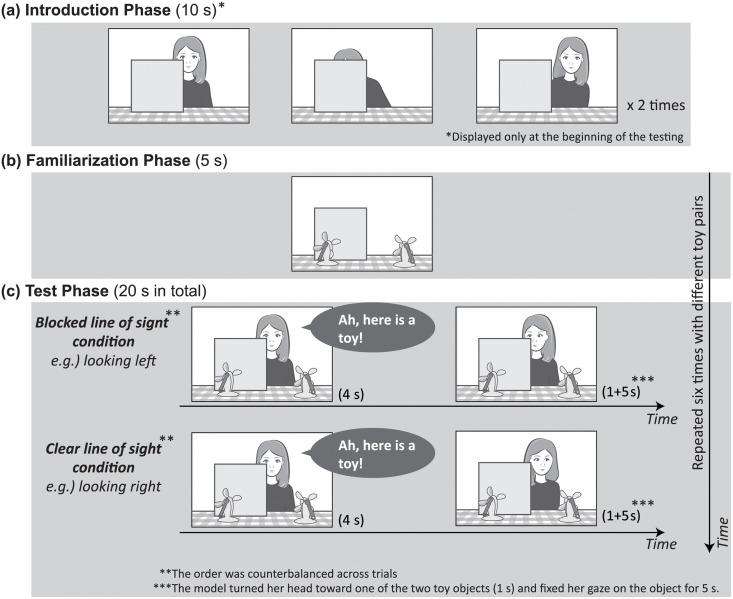
Schematic of study events. (**a**) Introduction Phase (10 s): The female model moved to the left and then to the right twice behind the barrier. This served to make infants aware that it occluded the model. (**b**) Familiarization Phase (5 s): Two identical toy objects were displayed with a barrier. One of the toy objects was placed in front of the barrier, and the other was not. (**c**) Test Phase (20 s in total): The model stared straight ahead for 4 s accompanied by a voice saying, “Ah, Omocha-ga Aruyo! (Ah, here is a toy!).” Then, the model gazed at either the toy object that was occluded by the barrier, (Blocked Line of Sight Condition) or the toy object that was not occluded by the barrier (Clear Line of Sight Condition). The order of the Blocked Line of Sight and Clear Line of Sight Conditions was counterbalanced. Note: An eye-catcher scene was displayed before the Introduction Phase and Familiarization Phase. It was also displayed before both the Blocked Line of Sight and Clear Line of Sight Conditions in the Test Phase, respectively. This was done to direct the infants’ visual attention toward the screen.

The stimulus started with a scene with a female model seated behind a barrier. The model then moved leftward or rightward twice alternatively for 10 s to make the infants aware that a barrier occluded the model (Fig 1a). The Introduction Phase was displayed once at the beginning of the experiment.

#### Familiarization Phase

Two identical toy objects were placed on each side of the table. A toy object with a barrier and another toy object without a barrier were displayed simultaneously for 5 s. In this phase, the model was not present (Fig 1b).

#### Test Phase

A female model appeared again on the other side of the table behind the toy objects; as in the Familiarization Phase, a toy object with a barrier and another toy object without a barrier were displayed simultaneously. The model stared straight ahead for 4 s accompanied by a voice saying, “Ah, Omocha-ga Aruyo! (Ah, here is a toy!).” This was done because although infants in both age groups could not understand the meaning of the utterance, they seemed to have the capacity to understand some abstract communicative functions of speech by the model [45, 46]. Moreover, we presented auditory stimuli in an infant-directed manner to attract their visual attention toward the screen [26, 47].

**Fig 2:**
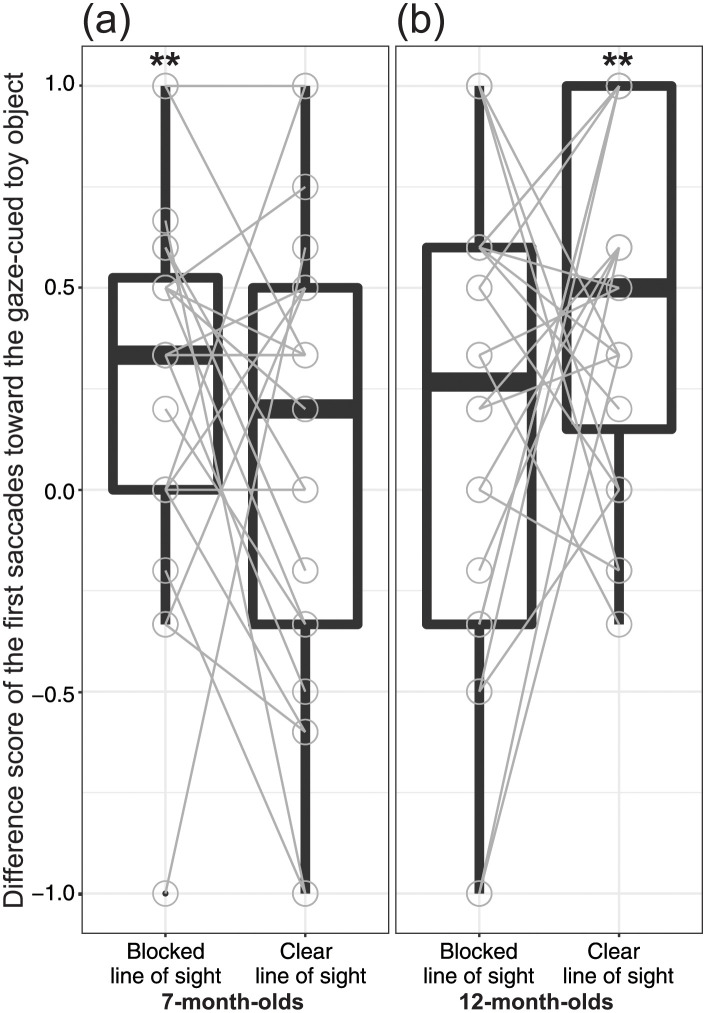
Difference score of the first saccades toward the gaze-cued toy object for the Blocked Line of Sight and Clear Line of Sight Conditions. Results are shown for the (a) 7-month-old infants and (b) 12-month-old infants. The central mark indicates the median, and the bottom and top edges of the box indicate the 25th and 75th percentiles, respectively. The upper whisker (vertical line) extends from the third quartile to the largest value. However, this is no further than 1.5 interquartile ranges from the third quartile. The lower whisker extends from the 25th percentile down to the smallest value. This goes no further than 1.5 interquartile ranges from the first quartile. **p < 0.01.

The model then turned her head toward one of the two toy objects (1 s) and fixed her gaze on the object for 5 s. For the Blocked Line of Sight Condition, the model turned her head right (or left) toward a toy object placed in front of the barrier, which would be invisible to the model. For the Clear Line of Sight Condition, the model turned her head right (or left) toward a toy object that was not occluded by the barrier, which would be visible to the model (Fig 2c).

In each trial, the Familiarization Phase (5 s) was followed by a Blocked Line of Sight Condition (10 s) and a Clear Line of Sight Condition (10 s). Therefore, a total of six Familiarization Phases plus 12 Test trials were displayed (six trials each for the Blocked Line of Sight and Clear Line of Sight Conditions); the total duration was approximately 160 s. The orders of the Blocked Line of Sight and Clear Line of Sight Conditions were counterbalanced across trials. The order of presentation of the toy objects was also counterbalanced among the participants. An eye-catcher was displayed before the Introduction Phase, the Familiarization Phase, and a Blocked Line of Sight and Clear Line of Sight trials in the Test Phase, respectively, to attract the infants’ visual attention towards the screen. The videos were edited with Premiere Element 15.0 to control the duration of each phase. As the Introduction Phase was displayed once at the beginning of the experiment (10 s), the testing lasted approximately 3 min.

### Analysis

#### Introduction Phase and Familiarization Phase

The viewing duration for the entire display was analyzed.

#### Test Phase

We first defined three areas of interest (AOIs): the model’s face, a gaze-cued toy object, and a gaze-uncued toy object. We defined the first face-object saccade occurring during the 6 s period involving the model’s eye movement and gaze at the toy as the first saccade. The first saccade was formulated as the infant’s first eye movement shifted from the AOI of the model’s face towards either the gaze-cued (congruent saccade) or the gaze-uncued (incongruent saccade) toy object. This is after the model directed her gaze toward one of the two toys. The difference score of the first saccade was calculated by summing the number of trials with an incongruent saccade (-1) and the number of trials with a congruent saccade (+1) and dividing the result by the total available number of trials with face-to-object saccades [32, 48]. The interrater agreement of the first saccade was assessed by two observers for 25% of the data (Cohen’s κ = .92). A parametric test was performed when the relevant assumptions were met.

We analyzed infants who satisfied the following criteria: (1) infants who looked at the screen for more than 50% of the time during the Introduction and Familiarization Phases and (2) when we obtained at least three valid trials for each condition (at least six valid trials per infant). A valid trial was defined as one in which infants looked at the entire display for more than 50% of the time and when infants elicited a face-to-object saccade during the Test Phase.

In addition to the first saccade measure reported in the present study, we further performed exploratory analyses of the proportion of the first looking time [42] and the proportion of the total looking time [27]. The proportion of the first looking time measure [42] was calculated by dividing the duration of the first looking at the gazed-cued object by the total duration of the first looking at both the gaze-cued and gaze-uncued objects. This is because it is likely that the proportion of the first looking time reflects not only spontaneous attentional orienting but also attention holding, which is related to infants’ inspection of the gaze-cued toy object [19]. Moreover, the proportion of the first looking time reflects the output of cognitive processing based on the preceding cue. This is not affected by the search process in the next 5 s when the model gazed at a toy object. In addition to the proportion of the first looking time, we calculated the proportion of the total looking time [27] by dividing the duration of the looking time at the gaze-cued objects for 5 s by the total duration of the total looking at both the gaze-cued and gaze-uncued objects while the model fixated on the object. This measure reflects the search process for 5 s, while the model gazed at a toy object.

### Results

Regarding the number of valid trials [7-month-olds (Blocked Line of Sight): *M* = 4.5, *SD* = 1.2; 7-month-olds (Clear Line of Sight): *M* = 4.2, *SD* = 1.0; 12-month-olds (Blocked Line of Sight): *M* = 4.5, *SD* = 1.1; 12-month-olds (Clear Line of Sight): *M* = 4.4, *SD* = 1.0], we applied a two-way mixed-design analysis of variance (ANOVA) with group (7-month-old versus 12-month-old infants) used as a between-subject factor and condition (Blocked Line of Sight versus Clear Line of Sight) used as a within-subject factor. However, we did not find any significant main effects [group: *F*(1, 46) = 0.10, *p* = 0.76, *η*_*p*_^*2*^ = 0.002; condition: *F*(1, 46) = 1.28, *p* = 0.26, *η*_*p*_^*2*^ = 0.03] or interaction [*F*(1, 46) = 0.72, *p* = 0.40, *η*_*p*_^*2*^ = 0.02].

### Introduction Phase

Infants in both groups looked at the display most of the time during the Introduction Phase (10 s), and the viewing duration was not significantly different across groups [7-month-olds: *M* = 9.08 s, *SD* = 1.23; 12-month-olds: *M* = 9.60 s, *SD* = 0.67, *Z* = 1.20, *p* = 0.23].

### Familiarization Phase

Infants in both groups looked at the display most of the time in the Familiarization Phase (5 s), and the viewing duration was not significantly different across groups [7-month-olds: *M* = 4.10 s, *SD* = 0.57; 12-month-olds: *M* = 4.21 s, *SD* = 0.33, *Z* = 0.43, *p* = 0.67].

### Test Phase

#### First saccade

Shapiro-Wilk tests were conducted for the data by condition and age group. The results indicated that all distributions were normal except for the Clear Line of Sight Condition among the 12-month-old infants. Therefore, we applied a non-parametric Wilcoxon test to the Clear Line of Sight Condition for the 12-month-old infants.

For 7-month-old infants (Fig 2a), the one-sample *t*-test showed that the difference score of the first saccade for the Blocked Line of Sight Condition was significantly higher than the chance level [*M*_Blocked line of sight_ = 0.28, *SD* = 0.45; *t*(23) = 3.00, *p* = 0.006, *d* = 0.61]. However, the one-sample *t*-test examining the difference score of the first saccade for the Clear Line of Sight Condition showed that scores was not significantly higher than chance level [*M*_Clear line of sight_ = 0.08, *SD* = 0.56; *t*(23) = 0.66, *p* = 0.52, *d* = 0.13].

For 12-month-old infants (Fig 2b), the one-sample *t*-test showed that the difference score of the first saccade for the Blocked Line of Sight Condition was not significantly higher than chance level [*M*_Blocked line of sight_ = 0.16, *SD* = 0.58; *t*(23) = 1.35, *p* = 0.19]. In contrast, non-parametric Wilcoxon test revealed that the difference score of the first saccade for the Clear Line of Sight Condition was significantly higher than chance level [*M*_Clear line of sight_ = 0.47, *SD* = 0.44; *Z* = 3.69, *p* < 0.001].

We further applied a two-way mixed-design ANOVA to the difference scores of the first saccade, with group (7-month-old versus 12-month-old infants) and condition (Blocked Line of Sight versus Clear Line of Sight) used as between- and within-subject factors, respectively. The main effects of group [*F*(1,46) = 1.95, *p* = 0.17, *η*_*p*_^*2*^ = 0.04] and condition [*F*(1,46) = 0.22, *p* = 0.64, *η*_*p*_^*2*^ = 0.004] were not significant. The two-way interaction of group × condition was significant [*F*(1,46) = 5.24, *p* = 0.03, *η*_*p*_^*2*^ = 0.10]. Subsequent analysis revealed that the difference scores of the first saccade in the 12-month-old infants were significantly higher than those in the 7-month-old infants in the Clear Line of Sight Condition [*F*(1,46) = 7.20, *p* < 0.01, *η*_*p*_^*2*^ = 0.14]. However, the difference score of the first saccade in the 12-month-old infants was not significantly different from that in the 7-month-old infants in the Blocked Line of Sight Condition [*F*(1,46) = 0.59, *p* = 0.45, *η*_*p*_^*2*^ = 0.01]. Moreover, the difference score of the first saccade in the Blocked Line of Sight Condition was not significantly different from that in the Clear Line of Sight Condition in both 7-month-old [*F*(1,23) = 1.94, *p* = 0.18, *η*_*p*_^*2*^ = 0.08] and 12-month-old infants [*F*(1, 23) = 3.31, *p* = 0.08, *η*_*p*_^*2*^ = 0.13].

#### Proportion of the first looking time

**Fig 3:**
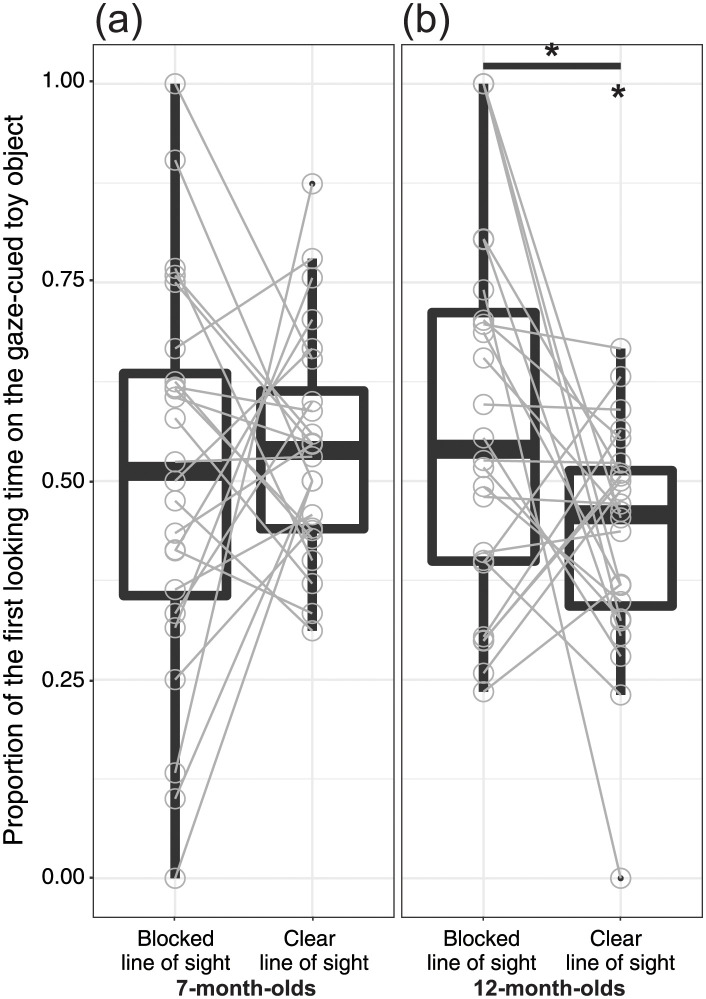
Proportion of the first looking time on the gaze-cued toy object for the Blocked Line of Sight and Clear Line of Sight Conditions. Results are shown for the (a) 7-month-old infants and (b) 12-month-old infants. The central mark indicates the median, and the bottom and top edges of the box indicate the 25th and 75th percentiles, respectively. The upper whisker (vertical line) extends from the third quartile to the largest value. However, this was no further than the 1.5 interquartile ranges from the third quartile. The lower whisker extends from the 25th percentile down to the smallest value. However, this was no further than 1.5 interquartile ranges from the first quartile. * *p* < 0.05.

For the 7-month-old infants (Fig 3a), the proportions of the first looking time for the gaze-cued toy object in both Blocked Line of Sight [*M*_Blocked line of sight_ = 0.51, *SD* = 0.25; *t*(23) = 0.12, *p* = 0.91] and Clear Line of Sight conditions [*M*_Clear line of sight_ = 0.54, *SD* = 0.14; *t*(23) = 1.48, *p* = 0.15] were not significantly different compared to the chance level. As for 12-month-old infants (Fig 3b), the proportion of the first looking time for the gaze-cued toy object was significantly below the chance level in the Clear Line of Sight Condition [*M*_Clear line of sight_ = 0.43, *SD* = 0.15; *t*(23) = 2.41, *p* = 0.02, *d* = 0.49], but not significantly different from the chance level in the Blocked Line of Sight Condition [*M*_Blocked line of sight_ = 0.58, *SD* = 0.24; *t*(23) = 1.61, *p* = 0.12].

We further applied a two-way mixed-design ANOVA to the proportions of the first looking time, with group (7-month-old versus 12-month-old infants) and condition (Blocked Line of Sight versus Clear Line of Sight) used as between- and within-subject factors, respectively. The main effects of group [*F*(1,46) = 0.33, *p* = 0.57, *η*_*p*_^*2*^ = 0.01] and condition [*F*(1,46) = 1.69, *p* = 0.20, *η*_*p*_^*2*^ = 0.04] were not significant. The two-way interaction of group × condition was significant [*F*(1,46) = 4.63, *p* = 0.04, *η*_*p*_^*2*^ = 0.09]. Subsequent analysis revealed that the proportion of the first looking time in the 7-month-old infants was significantly higher than that in the 12-month-old infants for the Clear Line of Sight Condition [*F*(1,46) = 7.61, *p* < 0.01, *η*_*p*_^*2*^ = 0.14]. Moreover, the proportion of the first looking time in the Blocked Line of Sight Condition was significantly higher than that in the Clear Line of Sight Condition in 12-month-old infants [*F*(1,23) = 5.84, *p* = 0.02, *η*_*p*_^*2*^ = 0.21]. However, there was no significant difference in the proportion of the first looking time between the 7-month-old and 12-month-old infants in the Blocked Line of Sight Condition [*F*(1,46) = 1.05, *p* = 0.31, *η*_*p*_^*2*^ = 0.02]. Moreover, there was no significant difference in the proportion of the first looking time between the Clear Line of Sight Condition and the Blocked Line of Sight Condition in the 7-month-old infants [*F*(1,23) = 0.37, *p* = 0.55, *η*_*p*_^*2*^ = 0.02].

#### Proportion of the total looking time

**Fig 4:**
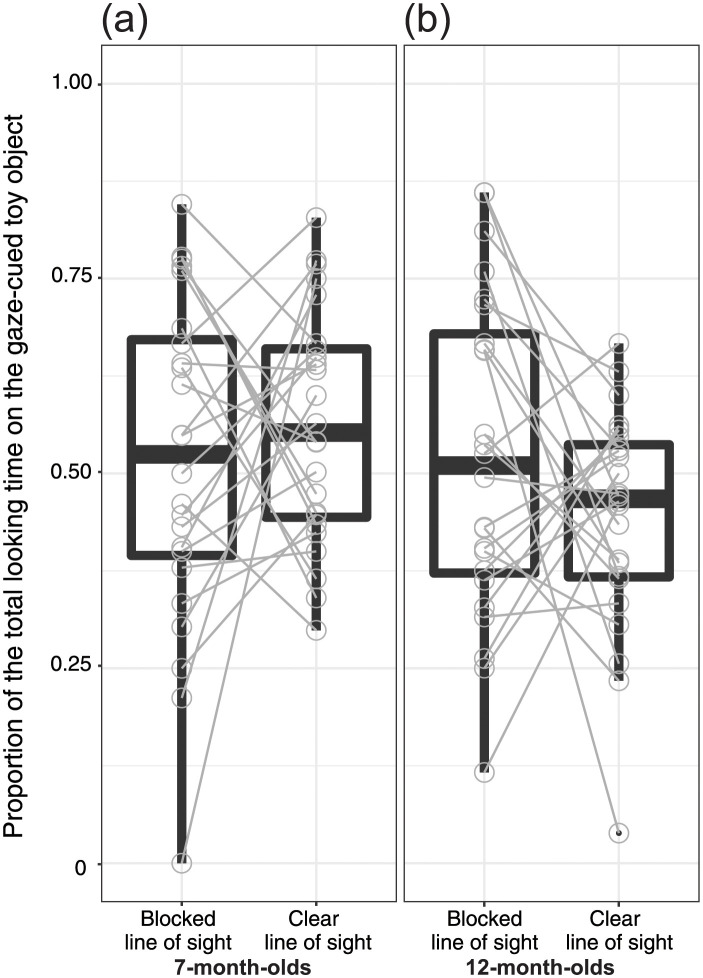
Proportion of the total looking time on the gaze-cued toy object for the Blocked Line of Sight and Clear Line of Sight Conditions. Results are shown for the (a) 7-month-old infants and (b) 12-month-old infants. The central mark indicates the median. The bottom and top edges of the box indicate the 25th and 75th percentiles, respectively. The upper whisker (vertical line) extends from the third quartile to the largest value. However, this was no further than 1.5 interquartile ranges from the third quartile. The lower whisker extends from the 25th percentile down to the smallest value. However, this was no further than 1.5 interquartile ranges from the first quartile.

For the 7-month-old infants (Fig 4a), the proportions of the total looking time for the gaze-cued toy object in both the Blocked Line of Sight [*M*_Blocked line of sight_ = 0.52, *SD* = 0.21; *t*(23) = 0.37, *p* = 0.71] and Clear Line of Sight [*M*_Clear line of sight_ = 0.56, *SD* = 0.15; *t*(23) = 2.00, *p* = 0.06] conditions were not significantly different, as compared to the chance level. As for the 12-month-old infants (Fig 4b), the proportions of the total looking time in the Blocked Line of Sight [*M*_Blocked line of sight_ = 0.52, *SD* = 0.21; *t*(23) = 0.49, *p* = 0.63] and Clear Line of Sight [*M*_Clear line of sight_ = 0.44, *SD* = 0.14; *t*(23) = 1.97, *p* = 0.06] conditions were not significantly different from the chance level.

We further applied a two-way mixed-design ANOVA to the proportions of the total looking time for group (7-month-old versus 12-month-old infants) and condition (Blocked Line of Sight versus Clear Line of Sight) used as between- and within-subject factors, respectively. We did not find any significant main effects of group [*F*(1, 46) = 2.84, *p* = 0.10, *η*_*p*_^*2*^ = 0.06] or condition [*F*(1, 46) = 0.17, *p* = 0.68, *η*_*p*_^*2*^ = 0.003] or their interaction [*F*(1, 46) = 2.42, *p* = 0.13, *η*_*p*_^*2*^ = 0.05].

## Discussion

The current study demonstrated that 12-month-old infants followed the model’s gaze towards a gaze-cued toy object, which the model could see, while the 7-month-old infants did not. Moreover, the exploratory analysis revealed that the proportion of the first looking time on the gaze-cued toy object with a barrier was significantly higher than that on the gaze-cued toy object without a barrier in 12-month-old infants, but not in 7-month-old infants. However, no significant effects were found for the proportion of the total looking time. The significant difference in the first saccade was not simply attributed to the looking duration of the Introduction, Familiarization, and Test Phases or the number of valid trials because we did not find any significant group differences for these measurements. Thus, in the current research, the 12- month-old infants could understand the visual experience of the model, but the 7-month-old infants showed no evidence of such capacity.

The current findings can advance the existing literature on VPT1 studies. Most previous studies have tested infants aged over 12 months and have reported that the capacity of VPT1 emerged at around 14 months, using the head-turning paradigm and looking time as measurements [16, 20, 21]. Although several previous studies have explored the abilities related to VPT1, such as the ability to compute beliefs, referential looking, and the visual experiences of others among the 7- and 12-month-old infants [23–25, 41], their paradigms did not directly test VPT1 abilities based on the definition of VPT1 [7]. For example, Moll and Tomasello [41] demonstrated that even 12-month-old infants locomoted toward a location that an adult had looked at but that was hidden from infants by an occluding object, such as a wall or a drawer. Importantly, the experimental paradigm did not manipulate the visual experience of the model but manipulated the infant’s own visual experience, that is, the experimental paradigm seemed to trigger the infant’s referential expectations for the viewing behavior of the adult. Therefore, it is important to directly test whether young infants can compute others’ visual experiences with VPT1 processing. Unlike the study by Moll and Tomasello [41], we directly tested how gaze-following performance can be modulated by VPT1 abilities in 7- and 12-month-old infants, similar to previous studies that used the head-turning paradigm [20, 21]. By doing so, we demonstrated that 12-month-old infants seem to have the capacity to compute others’ visual experiences.

Although we found a significant effect of the first saccade proportion against the chance level on the Blocked Line of Sight Condition in 7-month-old infants and on the Clear Line of Sight Condition in 12-month-old infants, we did not observe such a significant difference across the conditions in each age group. It is possible that the gaze-following pattern for the Clear Line of Sight Condition in the 7-month-old infants may be counterintuitive, as the proportion of the first saccade towards the gaze-cued object in the Blocked Line of Sight Condition was significantly above chance; however, this was not the case in the Clear Line of Sight Condition. We presumed that the developmental changes in gaze-following between 7- and 12-month-old infants could be mediated by the different cognitive mechanisms underlying gaze-following involved in VPT1 computation. The 12-month-old infants directed their first gaze towards the gaze-cued toy object when the model could see the gaze-cued toy object but not when it was occluded by the barrier. This suggests that 12-month-old infants represented the model’s visual experience [36, 49], which was consistent with the idea that gaze-following can be processed with a rich interpretation [50]. Conversely, 7-month-old infants followed the model’s gaze when the gaze-cued toy object was occluded by the barrier from the model’s perspective. This might not be based solely on a reflective mechanism, which does not consider the visual experience of others, and a lean interpretation, such as a conditioning mechanism, would also be insufficient [50, 51]. Rather, another explanation, possibly involving an attentional mechanism, might be involved (see below).

As for the exploratory analysis of the proportions of the first looking time [19, 42] and total looking time [27], because our current experimental design adopted the conventional gaze-following paradigm, it can be assumed that the proportions of the first looking time and total looking time on the gaze-cued object would be higher than that on the gaze-uncued object. However, as we placed a barrier to manipulate the model’s line of sight, we presumed that if infants could compute the visual perspective of the model, they may show a surprise reaction (i.e., prolonged duration of the first looking time) to the unexpected event [43], such as the model looking at the toy object occluded by a barrier (Blocked Line of Sight Condition). The results showed a conditional difference in 12-month-old infants but not in 7-month-old infants. Although we confirmed the significant conditional differences in 12-month-old infants, the proportion of the first looking time in the Blocked Line of Sight Condition was not significantly different from chance level. Furthermore, we did not find any significant conditional difference in the proportion of the looking time in 7- and 12-month-old infants. As we presumed that 12-month-old infants might exhibit prolonged first looking time on the gaze-cued toy object in the Blocked Line of Sight Condition based on previous findings [43], this was somewhat unexpected.

The unexpected results for both the looking time and the first saccade may be due to several reasons. Regarding the looking time, our current experimental paradigm may not be suitable for measuring the violation of expectation, although we designed our experimental procedures based on a similar experimental paradigm that induced a prolonged looking time in an unexpected event [43]. Most conventional studies adopted a violation expectation experimental paradigm that measures the looking time until the infants get bored and demonstrated the possibility of their understanding of another person’s perspective; this was observed in 14-month-olds [16] and 3-month-olds [18]. Conversely, we adopted a short duration of the visual stimulus, which may be inadequate in inducing a prolonged looking time during unexpected gaze behavior (i.e., looking at a toy object behind a barrier). Second, we combined an expected and unexpected trial with a short duration in the test phase, which made it challenging to induce the violation of the expectation effect, unlike in a previous study [43]. Regarding the measure of looking behavior, the functional dissociation of the post-hoc measures (such as looking time towards an unexpected event) and online measures (such as the predictive gaze shift) have been reported [52]. Because predictive eye movements require not only the evaluation of the observed event but also an ongoing active processing—although not necessarily *conscious* processing—the decision about where to move the gaze occurs before the outcome of the event is perceived. Therefore, predictive eye movements need to consider multiple possible outcomes and choose the most appropriate outcome [52]. This finding is supported by several studies that showed that the prediction of action goals when measured online can occur later, at the age of 11 months, than when measured post hoc, such as looking time [22, 53, 54]. Since multiple cognitive processes should be involved in the predictive gaze shift, it is likely that the evidence of online computation of VPT1 was not observed until 12 months.

Gaze behaviors in 12-month-old infants may be explained in terms of congruency between the first saccade and the proportion of the first looking time. For the first saccade, the Clear Line of Sight Condition was congruent with the concept of seeing. Therefore, infants frequently followed the model’s gaze when the model looked at the toy object without a barrier. Conversely, for the proportion of the first looking time, when the model looked at the toy object with a barrier, infants sometimes showed unexpected behavior, which led to a prolonged looking time, as compared to when the model looked at a toy object without a barrier. However, gaze behavior in 7-month-old infants seems to be inconsistent with the congruency effect. We presumed that the 7-month-old infants’ gaze behavior may have been caused by a selective attention mechanism, wherein visual saliency is enhanced by placing a barrier between the model’s eyes and the toy object. When the model saw a toy object hidden by an obstacle, the 7-month-old infants may have followed their gaze more frequently than for a toy object without an obstacle because the obstacle was closer to the model’s gaze. Therefore, the obstacle may have played a role in enhancing the infants’ gaze-following behavior toward the toy object with a barrier. Moreover, in the current experimental settings, we placed a barrier behind the toy. This may lead to a higher cognitive load as compared to the previous conventional gaze-following paradigms that solely displayed the toy objects [26, 27]. This may cause inconsistent gaze behaviors in 7-month-old infants.

This study had some limitations. First, quite a few 7-month-old infants (*n* = 24) were withdrawn from the study compared to 12-month-old infants (*n* = 10). This was mainly because they did not meet the inclusion criteria for eye movements. The reason for the high number of withdrawals among the 7-month-olds was that our task consisted of several phases that were more demanding for younger infants. This was different from previous infants’ gaze-following studies; so, a less demanding task should be developed to reduce the number of withdrawn infants. Second, we found a prolonged first looking time in the Blocked Line of Sight Condition but not in the proportion of the total looking time, which was somewhat unexpected. This may be because the proportion of the first looking time can reflect the output of the cognitive processing based on the immediately preceding cue, which is unaffected by visual searches. Further studies must characterize the behavioral properties of the proportions of the first looking time and the total looking time.

In conclusion, by combining a robust phenomenon, such as gaze-following and experimental manipulation to examine VPT1 ability, we have revealed that 12-month-old infants, but not 7-month-old infants, followed an adult’s gaze based on the computation of the model’s line of sight. Our research is the first to demonstrate that 12-month-old infants have the capacity to compute VPT1 by introducing a gaze-following experimental setting.

## Supporting information

S1 File (XLSX)
